# Antifatigue Effects of the Aqueous Extracts of Myrtle Berries, Apple and Clove: An Animal Study

**DOI:** 10.5812/ijpr-140323

**Published:** 2023-11-11

**Authors:** Akram Alembagheri, Homa Hajimehdipoor, Mona Khoramjouy, Somayeh Esmaeili, Mehrdad Faizi

**Affiliations:** 1 Department of Traditional Pharmacy, School of Traditional Medicine, Shahid Beheshti University of Medical Sciences, Tehran, Iran; 2 Department of Traditional Pharmacy, Traditional Medicine and Materia Medica Research Center, School of Traditional Medicine, Shahid Beheshti University of Medical Sciences, Tehran, Iran; 3 Phytochemistry Research Center, Shahid Beheshti University of Medical Sciences, Tehran, Iran; 4 Department of Pharmacognosy, School of Pharmacy, Shahid Beheshti University of Medical Sciences, Tehran, Iran; 5 Department of Pharmacology and Toxicology, School of Pharmacy, Shahid Beheshti University of Medical Sciences, Tehran, Iran

**Keywords:** Fatigue, Persian Medicine, *Myrtus communis* L., Central Fatigue, Sleep Deprivation, Iranian Traditional Medicine, *Malus domestica* Borkh., *Syzygium aromaticum* (L.) Merr. & L. M. Perry

## Abstract

**Background:**

Fatigue is one of the most prevalent symptoms, increasing worldwide with no specific medication for fatigue. Iranian traditional medicine (ITM), or Persian medicine, is a reliable source for discovering natural medicine for diseases and their symptoms. *Myrtus communis* L. (Myrtle), *Malus domestica* Borkh. (Apple), and *Syzygium aromaticum* (L.) Merr. & L. M. Perry (Clove) have been utilized as brain and heart tonics in ITM. Based on ITM, cardiac tonics decrease fatigue by enhancing heart function and increasing blood flow to tissues. These plants, particularly myrtle berries, have been utilized as potent enlivening agents that reduce mental fatigue.

**Objectives:**

This study aims to investigate the effects of aqueous extracts of these plants on weight-loaded forced swimming (WLFS) tests and three doses of aqueous myrtle extract in an animal model of chronic sleep deprivation-induced fatigue.

**Methods:**

Five groups of rats (n = 6) were evaluated: Sham, control, apple-treated, clove-treated, and myrtle-treated groups. After 28 days of treatment, the WLFS test was performed, and swimming time was recorded. Subsequently, central fatigue was induced in rats by chronic sleep deprivation for 21 days. Five groups of rats (n = 6) were evaluated: Sham, control (sleep-deprived, which received water), and three sleep-deprived + treatment groups, which received aqueous myrtle extract (350, 700, and 1000 mg/kg). An open field test on the 20th day and a WLFS test on the 21st day were performed.

**Results:**

The myrtle berries significantly increased glucose, reduced lactate dehydrogenase (LDH) levels, and enhanced swimming time. Fatigue caused by chronic sleep deprivation increased malondialdehyde (MDA), tumor necrosis factor-α (TNF-α), interleukin-1β (IL-1β), and LDH while decreased superoxide dismutase (SOD), glucose, and swimming time. In all treatment groups, SOD levels and swimming time were increased, whereas MDA, IL-1β, and TNF-α levels were decreased significantly. Only the 1000 mg/kg dose significantly reduced LDH levels (P < 0.001). The treatment significantly improved the velocity and the total distance moved in the open-field test.

**Conclusions:**

According to the results, the myrtle berries reduced fatigue in two animal models, probably due to its phenolic compounds, flavonoids, and polysaccharides.

## 1. Background

In clinical medicine, fatigue is one of the most prevalent symptoms. It is described as difficulty initiating or continuing voluntary activities (1). In addition to making a person more excitable, fatigue affects their talents, motivation, concentration, memory, and social performance (2). It is a typical symptom in various disorders, including cancer, chronic heart failure, multiple sclerosis, sleep disorders, Parkinson's disease, and several endocrine diseases. Several systemic and neurological conditions can induce fatigue, but its precise etiology is still unknown in some patients (1). Two oxidative stress and exhaustion mechanisms can be considered effective in causing fatigue. The production and accumulation of free radicals cause oxidative stress and damage the body. According to the exhaustion theory, the accumulation of excess metabolites and depleted energy sources also produces fatigue (3). Furthermore, oxidative stress triggers inflammatory responses and elevates proinflammatory cytokines such as tumor necrosis factor-α (TNF-α) and interleukin-1β (IL-1β) (4). Chronic sleep deprivation, widespread in today's society, contributes to fatigue and even serious illnesses (5, 6). Sleep disturbance is believed to be a central fatigue inducer. Central fatigue is a neuromuscular disorder that is a common symptom of chronic fatigue (7). Although there is no specific medication for fatigue, many scientists are looking into medicinal plants and other natural remedies to accelerate the removal of fatigue-related metabolites and lessen fatigue (8, 9). Numerous plant substances, including triterpenoids, polysaccharides, and flavonoids, have been investigated to reduce the signs of fatigue. In addition to increasing physical strength, they also prevent the onset of fatigue and speed up its relief (9). Iranian traditional medicine (ITM), or Persian medicine, is a reliable resource for discovering natural remedies for illnesses and their symptoms. In ITM, *Myrtus communis* L. (Myrtle), *Malus domestica* Borkh. (Apple) and *Syzygium aromaticum* (L.) Merr. & L. M. Perry (Clove) have been used as brain and heart tonics to enhance the physiological functioning of organs and increase the resistance of the organs to pathological situations (10). Cardiac tonics reduce fatigue by improving heart function and increasing tissue blood flow. Smelling the fresh leaves of *M. communis* and the fruit of *M. domestica* has brain-boosting benefits. These plants, especially myrtle fruit, have also been used as potent enlivening agents for mental fatigue relief (10, 11).

## 2. Objectives

In this study, the weight-loaded forced swimming test's animal model was used to preliminary investigate the anti-fatigue effect of these three plants. Then, the effect of the most effective plant was evaluated in an animal model of chronic sleep deprivation-induced fatigue. The aqueous extract of the most effective was standardized by the high-performance liquid chromatography (HPLC) method.

## 3. Methods

### 3.1. Plant Material and Extract Preparation

*Syzygium aromaticum, M. communis* fruit, and *M. domestica* (yellow apple of Tehran province) were acquired from the market in Tehran, Iran, and identified in the herbarium of the Traditional Medicine and Materia Medica Research Center (TMRC), Shahid Beheshti University of Medical Sciences, Tehran, Iran. The herbal market sample (HMS) numbers for *S. aromaticum, M. communis* fruit, and *M. domestica* were HMS-561, HMS-562, and HMS-563, respectively. The plant name has been checked with http://www.theplantlist.org. Maceration or decoction processes were employed for extraction in accordance with the preparation techniques for various medicinal dosage forms of all three plants in ITM references (10, 12). The aqueous clove extract was made using the three-day maceration method (plant/water ratio 1: 20 w/v). The extract was dried by rotary evaporator and freeze dryer. The myrtle fruits were extracted by decoction method with water (plant/water ratio 1: 20 w/v) for 120 min. The obtained aqueous extract of myrtle fruits was filtered to remove the fruit masses before evaporating and drying at 70°C in an oven. Apple juice was first made from apples using a juicer. Apple juice was concentrated using boiling and dried by a vacuum dryer oven. Each of these three aqueous extracts was dissolved in distilled water for administration in rats.

### 3.2. Ethical Considerations

The Ethics Committee of Shahid Beheshti University of Medical Sciences approved the proposal for the research (code: IR.SBMU.RETECH.REC.1399.1129, 2021). The in vivo part of the study was carried out following the NIH Animal Care and Use Committee Guide for the Care and Use of Laboratory Animals.

### 3.3. Chemicals and Kits

Acetonitrile HPLC grade (J.T.Baker®, USA), methanol HPLC grade (Samchun chemical company, Korea), formic acid (Merck, Germany), and gallic acid (Sigma-Aldrich, Germany) were used. Serum IL-1β levels were measured using an ELISA Kit (Catalog number: RLB00, R&D, USA), and serum TNF-α levels were measured using an ELISA Kit (Catalog number: RTA00 R&D, USA). Serum SOD levels were measured using an ELISA kit (Catalog number: NS-15032, Navandsalamat, Urmia, Iran). Serum MDA levels were measured using an ELISA kit (Catalog number: TPR-MDA96T, Teb Pazhouhan Razi Company kit, Tehran, Iran). Serum LDH levels were measured using an assay kit (Catalog number: BXC0242, Biorexfars, Fars, Iran). Serum glucose levels were measured using an assay kit (Catalog number: BXC0101, Biorexfars, Fars, Iran).

### 3.4. Investigation of Acute Oral Toxicity and Determination of LD50

An acute oral toxicity test was conducted based on OECD guidelines for the clove and myrtle fruit (13). In this manner, three female rats with identical weights were chosen for each plant, with a weight difference of no more than 20%. The rats had only access to water the previous night and had to fast. The following day, 2000 mg/kg of aqueous extracts of clove and myrtle fruit were administered by gavage. The rats were denied access to food for the following three hours, and their conditions were observed for 48 hours while the signs were checked. The toxicity test was repeated with a dose of 2000 mg/kg for three rats if all rats survived or if only one rat in a group died, following OECD guidelines. If needed, the acute toxicity test with a dose of 5000 mg/kg was also performed (14).

### 3.5. Animals

Sprague-Dawley male rats weighing 200 - 250 g and aged 6 - 8 weeks were obtained from TMRC. Animals were housed in temperature-controlled rooms (22°C) with 12-hour cycles of light and darkness and humidity of 45 - 65%. The rats were given free access to food and water (a standard diet).

### 3.6. Weight-loaded Forced Swimming Test

The male rats were divided into five groups (six rats in each group) to study the anti-fatigue effects of the extracts. The groups were as follows:

(1) The sham group which received distilled water orally.

(2) The control group was administered distilled water, and the weight-loaded forced swimming test was performed.

(3) The group administered the aqueous myrtle extract at 700 mg/kg and performed the weight-loaded forced swimming test.

(4) The group administered the aqueous apple extract at 2330 mg/kg and performed the weight-loaded forced swimming test.

(5) The group administered the aqueous clove extract at a dose of 333 mg/kg and performed the weight-loaded forced swimming test.

The samples were then administered via daily gavage over a duration of 28 days (a certain dose was determined within the range of doses included in traditional medicine references considering the LD_50_s) (10). A weight-loaded forced swimming test was conducted after 28 days (15, 16). In this manner, a load comprising 10% of the rat’s weight was attached to its tails. Each rat was then placed inside a water cylinder, and the swimming time was recorded until the point at which it was unable to return to the surface within 10 seconds. According to the standard working method with laboratory animals, the rats underwent CO_2_ anesthesia after an hour. The cardiac blood samples were collected to measure lactate dehydrogenase (LDH) levels, tumor necrosis factor-α (TNF-α), and glucose in the serum.

### 3.7. Induction of Chronic Sleep Deprivation

The modified multiple platform method (MMPM) was used to induce sleep deprivation for 21 days (6, 17). This was achieved by placing each group of rats in a 110×60×40 cm polyethylene water tank with 15 circular plastic platforms (6.5 cm in diameter). The tanks were filled with water at temperatures ranging from 20 to 25°C to a depth of one centimeter below the surface of the platforms. Rats were put back in their cages the following day after spending 14 hours per day in the tank, from 6:00 p.m. to 08:00 a.m. The rats were divided into five groups, each with six rats, including sham, sleep-deprived (control), and the three sleep-deprived + treatment groups. Treatment groups received 350, 700, and 1000 mg/kg of the aqueous myrtle extract daily via oral gavage for 21 days. A similar volume of water was given to other groups. An open-field behavioral test was conducted on the 20th day. On the final day, one hour following the gavage of the final aqueous extract, a weight-loaded forced swimming test was performed, as stated in section 3.6. According to the standard working method with laboratory animals, the rats underwent CO_2_ anesthesia after an hour. The cardiac blood samples were collected to measure the serum levels of LDH, TNF-α, glucose, malondialdehyde (MDA), superoxide dismutase (SOD), and interleukin-1β (IL-1β).

### 3.8. Open Field Test

The open field arena (60×60×60 cm) has black walls and floor. Each rat was placed in the center of the open field arena, and a digital camera placed above the apparatus was used to record the locomotor activity of the rats for ten minutes. Before each test, the test area was cleaned with 70% ethanol. Ethovision XT software (Noldus, The Netherlands) was used to analyze each recorded video (18).

### 3.9. Statistical Analysis

Using a one-way analysis of variance, the results of the various groups were analyzed, and the Tukey-Kramer multiple comparison test, was employed as a post-hoc test. A statistical difference was considered significant if P < 0.05. The data were shown as mean ± SEM.

### 3.10. High-Performance Liquid Chromatography (HPLC)

HPLC was done by the Shimadzu system equipped with a vacuum degasser and photodiode array detector. The spectrophotometric detection was performed at 280 nm. LabSolutions software was used for instrument control, collection, and processing. The column was C18 (4.6 × 250 mm, 5 µm). The flow rate was 1 mL/min, and the injection volume for all samples and standard solutions was 20 µL. The gradient program of the mobile phase is demonstrated in Table 1 (19).

**Table 1.** The Gradient Program of the Mobile Phase

Time, Min	% A, H_2_O + 0.1 % Formic Acid	% B, H_2_O + Acetonitrile (50: 50 v/v) + 0.1 % Formic Acid
**0**	95	5
**2**	95	5
**5**	55	45
**20**	0	100
**25**	0	100
**26**	95	5
**28**	95	5

### 3.11. Standard and Sample Preparation

Four different concentrations of gallic acid in water (0.001, 0.04, 0.2, and 1 mg/mL) were prepared. Subsequently, 200 mg of dried extract was transferred to a 10 mL volumetric flask and dissolved in water to volume. Filtration of the final solution was done using a membrane filter (0.45 µm). Sample and standard solutions were injected into the HPLC system three times, and gallic acid content in the aqueous extract was calculated using the area under the curve (AUC) of standard and sample peaks of gallic acid in the chromatograms.

## 4. Results

### 4.1. Preparation of Aqueous Plant Extract

The aqueous extracts of the plants were prepared. The yield percentages of the aqueous extract of the myrtle fruit, clove, and apple were 16.9, 15.7 and 5.8%, respectively.

### 4.2. Investigation of Acute Toxicity and Determination of LD50

Acute toxicity testing was carried out following OECD guidelines. The toxicity test was repeated with a dose of 2000 mg/kg for three rats after one died in the clove group, but all three rats lived in the myrtle group. Based on the number of living rats in each group, the LD_50_s were calculated. The clove group had an LD_50_ value of 2500 mg/kg, while the myrtle group had an LD_50_ value higher than 2000 mg/kg. Subsequently, the acute toxicity study was performed for the myrtle aqueous extract with a dose of 5000 mg/kg, and it had an LD_50_ value higher than 5000 mg/kg.

### 4.3. Effects of Aqueous Extracts on Swimming Time

**Figure 1.**
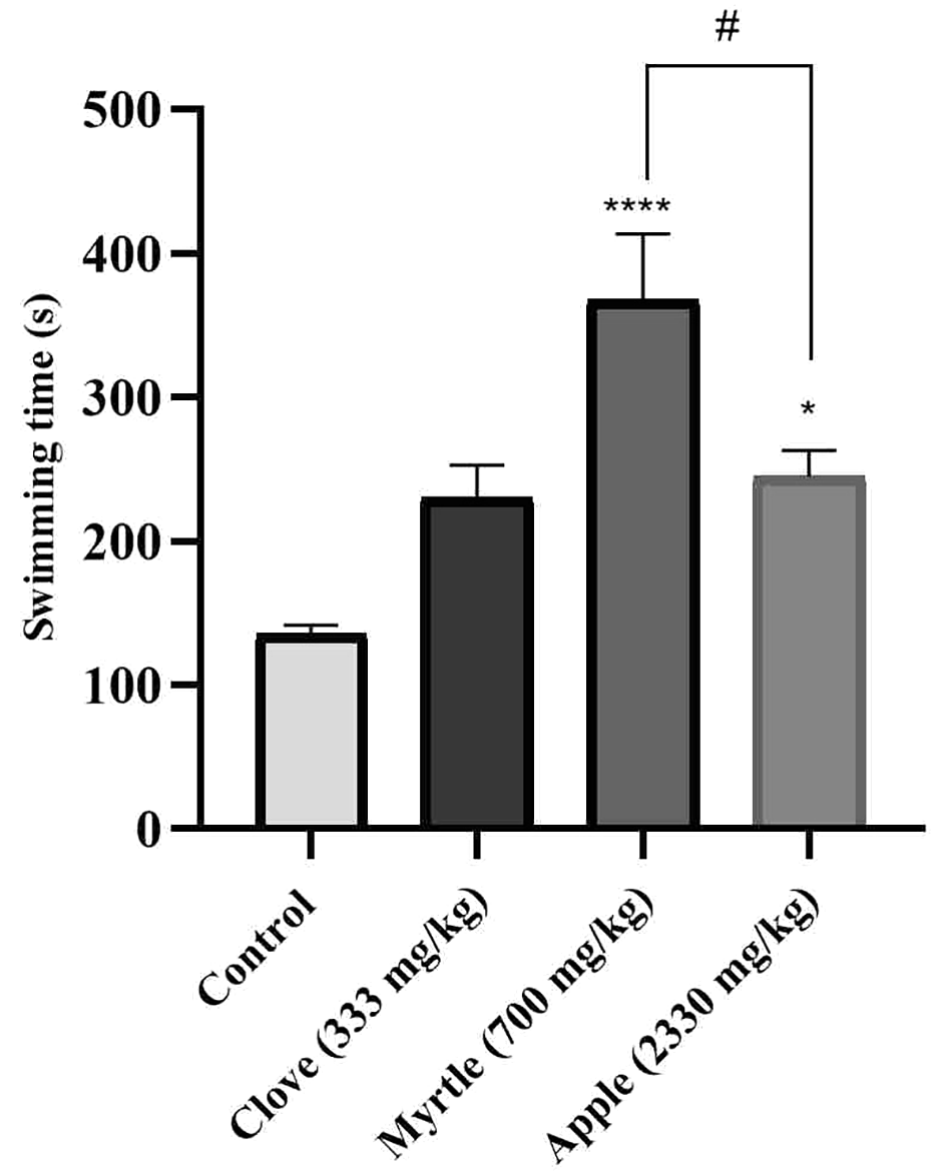
Effects of treatment with clove (333 mg/kg), myrtle (700 mg/kg), and apple (2330 mg/kg) on swimming time in weight-loaded forced swimming test. * P < 0.05 and **** P < 0.0001 compared to the control group. # P < 0.05 compared the apple group to the myrtle group. The results were obtained by one-way ANOVA followed by Tukey’s test. Data are described as mean ± SEM (n = 6).

The anti-fatigue effect of aqueous extracts of three plants was investigated using a weight-loaded forced swimming model. Compared to the control group, aqueous extracts of myrtle fruit and apple significantly increased the duration of forced swimming (Figure 1). 

### 4.4. Effects of Aqueous Extracts on LDH, TNF-α, and Glucose

**Figure 2.**
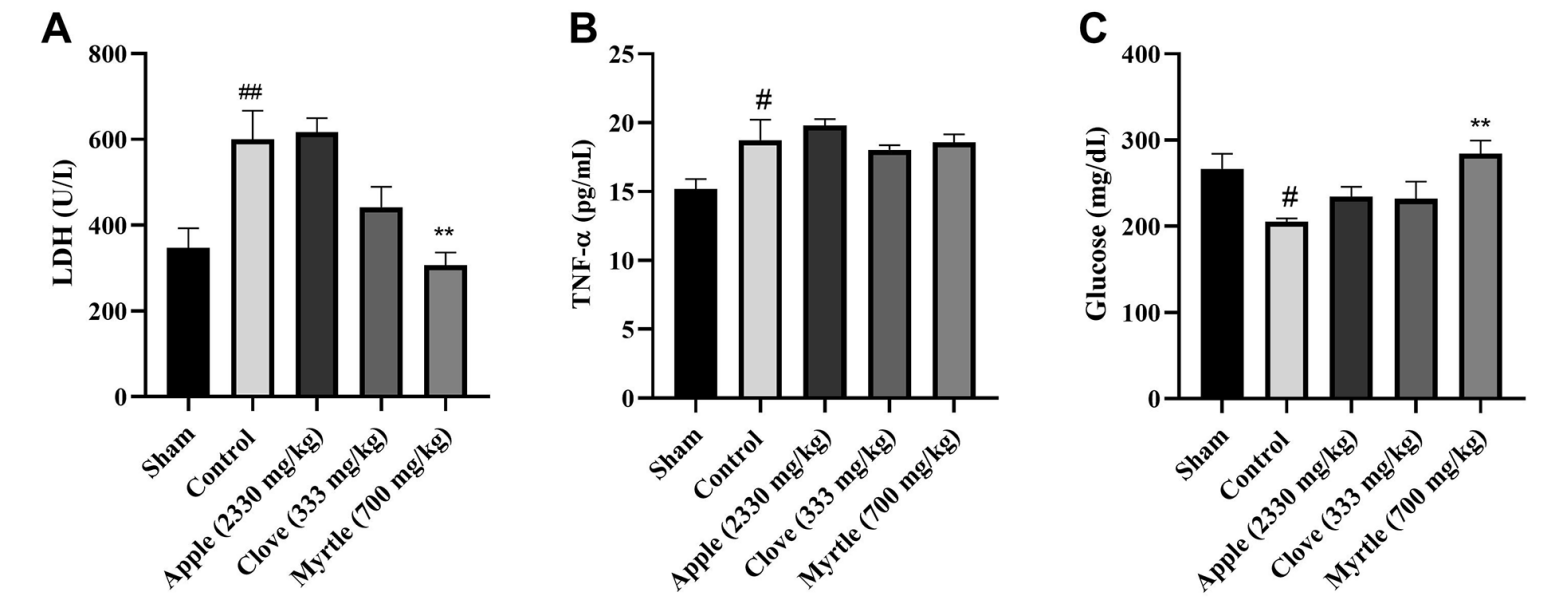
Effects of treatment with clove (333 mg/kg), myrtle (700 mg/kg), and apple (2330 mg/kg) on LDH, TNF-a, and glucose concentrations after 28 days of the experiment of weight-loaded forced swimming test. (A) LDH, (B) TNF-a, and (C) glucose. ** P < 0.01 compared to the control group. # P < 0.05 and ## P < 0.01 compared to the sham group. The results were analyzed by one-way ANOVA followed by Tukey’s test. Data are described as mean ± SEM (n = 6).

All three aqueous extracts increased the level of glucose compared to the control group, but this was significant (P < 0.01) only for aqueous myrtle extract (700 mg/kg). Both myrtle and clove aqueous extracts decreased the activity of lactate dehydrogenase (LDH), which was significant for aqueous myrtle extract as compared to the control group (P < 0.01). None of the three aqueous extracts could significantly change the tumor necrosis factor-α (TNF-α) level compared to the control group. The myrtle and clove aqueous extracts decreased the TNF-α level, but this was not significant (Figure 2). Based on the results, the aqueous myrtle extract performed best in lowering fatigue. In the following step, the animal model of chronic sleep deprivation-induced fatigue was used to evaluate the anti-fatigue benefits of myrtle.

### 4.5. Effects of Aqueous Myrtle Extract on swimming Time

**Figure 3.**
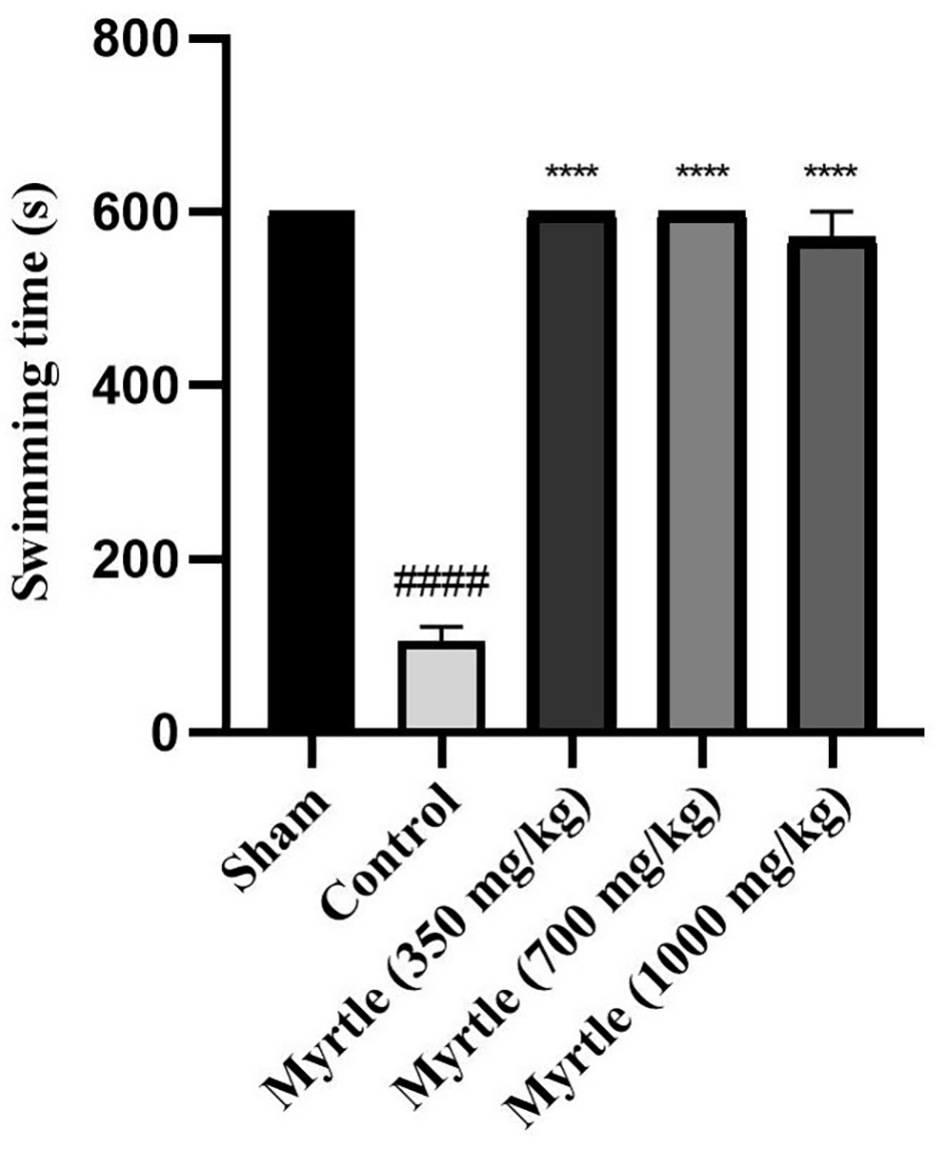
Effects of treatment with myrtle aqueous extract at doses of 350, 700, and 1000 mg/kg on swimming time in a weight-loaded forced swimming test after 21 days of the experiment. **** P < 0.0001 compared to the control group. #### P < 0.0001 compared to the sham group. The results were analyzed by one-way ANOVA followed by Tukey’s test. Data are described as mean ± SEM (n = 6).

After inducing sleep deprivation for 21 days using the modified multiple platform method (MMPM), on the 21st day, a weight-loaded forced swimming test was used to examine the anti-fatigue effects of 350, 700, and 1000 mg/kg doses of myrtle aqueous extract. In the control group, the swimming time was significantly decreased compared to the sham group (P < 0.0001). All three groups of aqueous extract significantly increased the duration of forced swimming compared to the control group (P < 0.0001, P < 0.0001, and P < 0.0001, respectively) (Figure 3). 

### 4.6. Effects of Aqueous Myrtle Extract on the Biochemical Parameters Related to Fatigue

**Figure 4.**
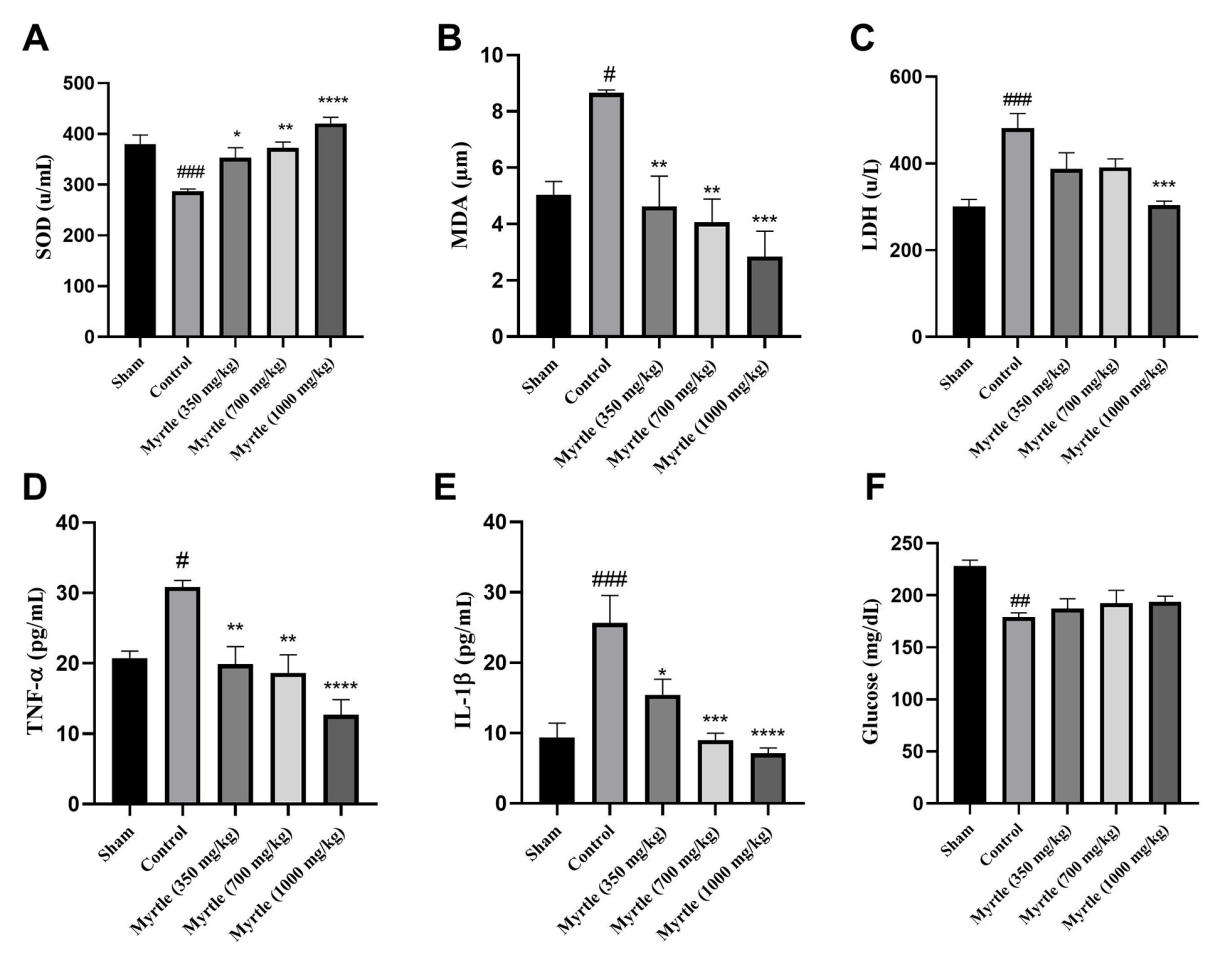
Effects of treatment with myrtle aqueous extract at doses of 350, 700, and 1000 mg/kg on SOD, MDA, LDH, TNF-a, IL-1β, and glucose concentrations after 21 days of chronic sleep deprivation-induced fatigue. (A) SOD, (B) MDA, (C) LDH, (D) TNF-α, (E) IL-1β, and (F) glucose. * P < 0.05, ** P < 0.01, *** P < 0.001 and **** P < 0.0001 compared to the control group. # P < 0.05, ## P < 0.01 and ### P < 0.001 compared to the sham group. The results were analyzed by one-way ANOVA followed by Tukey’s test. Data are described as mean ± SEM (n = 6).

As shown in Figure 4, the level of superoxide dismutase (SOD), LDH, TNF-α, glucose, malondialdehyde (MDA), and interleukin-1β (IL-1β) were measured in the serum of the rats that received daily doses of 350, 700 and 1000 mg/kg of myrtle extract for 21 days. Compared to the sham group, in the control group, SOD and glucose levels were significantly decreased (P < 0.001 and P < 0.001, respectively). Also, MDA, LDH, TNF-α, and IL-1β levels were increased significantly. In all the treatment groups, SOD levels were increased, and MDA levels were significantly decreased compared to the control group. LDH levels were decreased in all doses of the aqueous myrtle extract groups, but the reduction was significant only at the dose of 1000 mg/kg (P < 0.001). In the treatment groups, TNF-α levels were decreased significantly compared to the control group. The 1000 mg/kg group reduction was significantly greater than that of the other two doses (P < 0.0001). IL-1β levels decreased to different levels in a dose-dependent manner, which was significant for all three doses compared to the control group. In all three doses of aqueous extract, the glucose levels were not significantly changed compared to the control group (Figure 4). 

### 4.7. Effects of Aqueous Myrtle Extract on the Performance of the Rats in the Open-field Test

**Figure 5.**
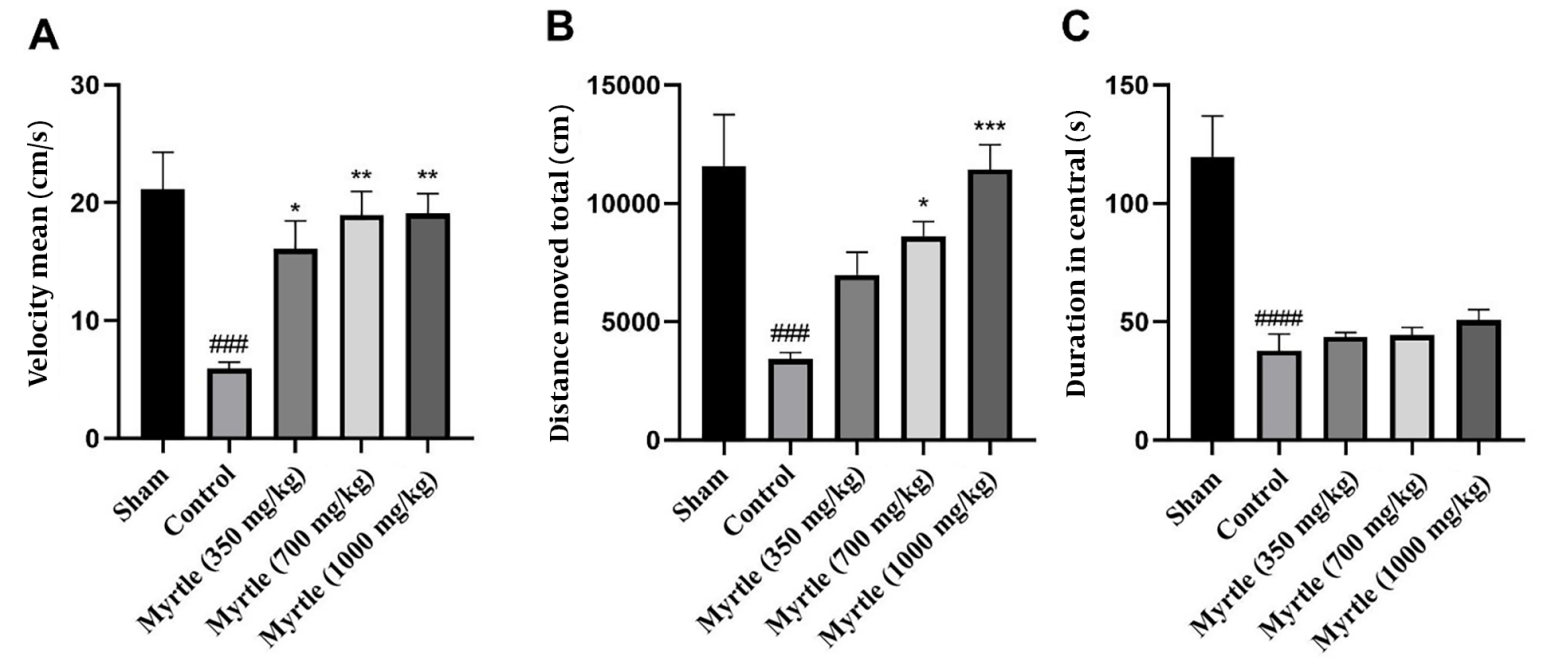
Effects of treatment with myrtle aqueous extract at doses of 350, 700, and 1000 mg/kg on velocity, total distance moved, and duration in central open field test after 21 days of the experiment. (A) Mean of velocity, (B) total distance moved, and (C) duration in central. * P < 0.05, ** P < 0.01 and *** P < 0.001 compared to the control group. ### P < 0.001 and #### P < 0.0001 compared to the sham group. The results were analyzed by one-way ANOVA followed by Tukey’s test. Data are described as mean ± SEM (n = 6).

For evaluating the performance of the rats in the open-field test, as shown in Figure 5, velocity, total distance moved, and duration in central were measured for all groups. In the control group, velocity, total distance moved, and the duration in the central zone were significantly decreased compared to the sham group (P < 0.001, P < 0.001, and P < 0.0001, respectively). In comparison to the control group, the velocity was increased by 350, 700, and 1000 mg/kg doses of the aqueous myrtle extract (P < 0.05, P < 0.01, and P < 0.01, respectively). The increase in the total distance moved was not significant at the dose 350 mg/kg group, while 700 and 1000 mg/kg doses significantly increased the distance (P < 0.05 and P < 0.001, respectively). There were no significant differences in duration in the central between the three doses compared to the control group.

### 4.8. High-performance Liquid Chromatography (HPLC)

**Figure 6.**
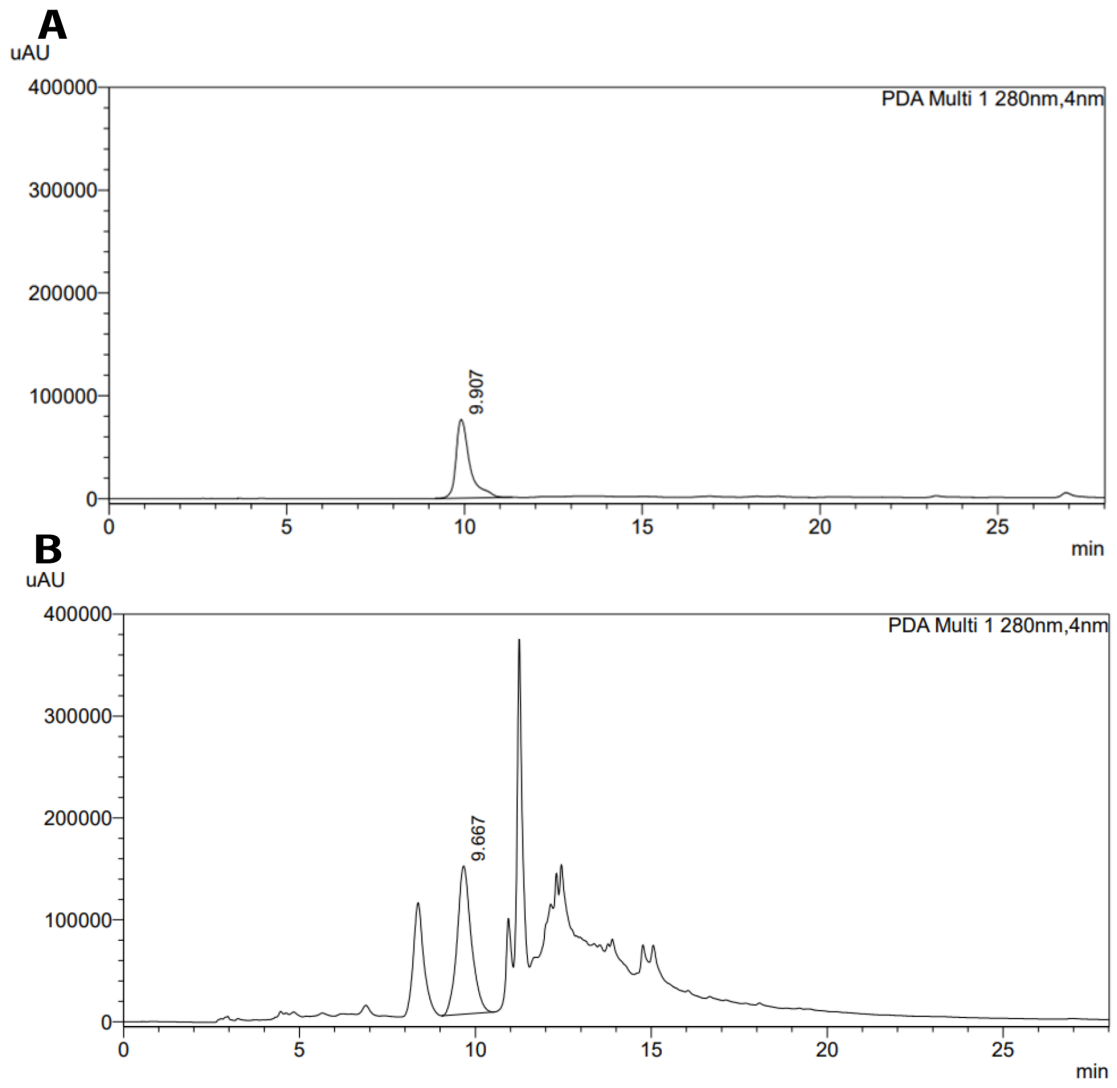
HPLC chromatogram of gallic acid (A) and myrtle aqueous extract (B)

The aqueous myrtle extract was chosen from three plants, and it was standardized using the HPLC method, which is a precise method for analyzing and standardizing extracts (20). The aqueous myrtle extract contains several compounds, such as gallic acid, ellagic acid, anthocyanins, and flavonols (21). We chose gallic acid to standardize the aqueous myrtle extract. The HPLC chromatograms of gallic acid standard solution and myrtle aqueous extract are presented in Figure 6. The gallic acid content of aqueous myrtle extract was 0.42%. Gallic acid standard calibration curve line equation was: y = 5647.54x - 7.49 (R^2^ = 1.00).

## 5. Discussion

Fatigue is one of the most prevalent issues in human societies with different intensity levels. Despite the lack of a specific medication for fatigue, the benefit of numerous herbal remedies for reducing fatigue in animal models has been described (15, 16). Aqueous *Moringa oleifera* Lam extract reduces fatigue by increasing body energy storage antioxidant capacity and decreasing lactic acid (15). *Panax ginseng* CA Mey reduces fatigue through several mechanisms, including improving mitochondrial function, enhancing the antioxidant system, and delaying the accumulation of metabolites (22).

In the current study, an animal model of the weight-loaded forced swimming test was used to evaluate the potential anti-fatigue effects of three aqueous extracts of myrtle, clove, and apple. Many factors can contribute to activity-related fatigue. A significant factor that leads to physical fatigue is a lack of energy. During exercise, energy reserves such as glycogen are consumed, and metabolic waste products, including lactic acid and ammonia, are produced. Glucose is primarily used to meet energy needs; ongoing exercise frequently causes hypoglycemia and reduces the ability to exercise continuously (23). In our study, the weight-loaded forced swimming decreased blood glucose levels in the control group, and the aqueous herbal extracts increased it, which was significant only for the myrtle aqueous extract (P < 0.01).

The accumulation of active oxygen species is a significant contributor to fatigue. During periods of high activity, the overproduction of active oxygen radicals causes oxidative stress, fatigue, and muscle damage, ultimately lowering a person's capacity for physical activity. LDH and creatine kinase (CK), two markers of muscle damage following intense exercise, are increased when physical fatigue develops (16). Our results showed that after the weight-loaded forced swimming test, serum LDH levels were higher in the control group compared to the sham group, which did not perform the swimming test. In the apple-treated group, LDH levels were similar to the control group, and the aqueous extract could not decrease LDH levels. However, in the clove and myrtle-treated groups, LDH levels were reduced compared to the control group, which was significant only for the aqueous myrtle extract. These observed effects can be due to phenolic compounds such as gallic acid in the myrtle aqueous extract. Previous studies indicated the effectiveness of gallic acid in reducing LDH in several animal models (24-27). In addition, the antioxidant features of myrtle fruit could effectively reduce oxidative stress (28, 29). The effectiveness of clove essential oil in lowering LDH levels has been demonstrated previously, which has been associated with the presence of eugenol and the antioxidant activity of essential oil. The primary component of clove oil is eugenol, which is highly soluble in organic solvents and slightly soluble in water. Therefore, the aqueous extract of clove contains a small amount of eugenol. As a result, the essential oil of clove can be more effective in lowering LDH levels than the aqueous extract of clove (30, 31).

The oxidative stress caused by exercise increases the production of inflammatory cytokines such as TNF-α, IL-1β, and IL-6 (32-35). Furthermore, it was observed that in the skeletal muscles of rats, acute exercise could trigger the nuclear factor (NF)-kappaB-TNF-α inflammatory signaling pathway (36, 37). Our results showed that TNF-α levels were higher in control and treatment groups after weight-loaded forced swimming than in the sham group. None of the extracts could significantly decrease TNF-α levels.

As shown in Figure 1, apple and myrtle aqueous extracts increased the forced swimming time. The swimming time was 2.7 times higher in the myrtle-treated group and 1.8 times higher in the apple-treated group than the control group. These results are consistent with those of earlier investigations. An aqueous extract of *Sonchus arvensis *L. has been shown to enhance exercise performance and lessen fatigue (38).

According to these results, the myrtle extract was most effective in reducing the fatigue indices among the three tested extracts. Therefore, this extract was selected for the next step. In the second part of our study, we subjected the rats to chronic sleep deprivation to induce fatigue by using the modified multiple platform method (MMPM). The study of Han et al. showed that various MMPM durations resulted in different brain and behavioral changes. Central fatigue could develop after the 21 days of MMPM (6).

Reduced physical activity, manifested in our study as diminished swimming time, is one of central fatigue's most obvious and direct effects (39, 40). Our results showed that fatigue produced by chronic sleep deprivation resulted in a significant decrease in swimming time in the control group compared to the sham group in the weight-loaded forced swimming test, commonly used to measure physical endurance in fatigue studies. In addition, all three doses of myrtle extract improved swimming time compared to the control group.

Central fatigue can cause physical exhaustion, which in turn can lead to a decrease in muscular strength. Lactate dehydrogenase lactate dehydrogenase and CK activities indicate how well muscles work (40). In our study, as compared with the sham group, sleep deprivation-induced fatigue elevated LDH levels in the control group. Only one of the myrtle aqueous extract doses (1000 mg/kg) reduced LDH level compared to the control group, demonstrating that it preserved muscular function during central fatigue.

Chronic sleep deprivation causes oxidative stress and affects the balance of the antioxidant system and oxidant. The body possesses an enzymatic defense system that comprises SOD, catalase (CAT), and glutathione peroxidase (GPx), which can deal with oxidative stress (20). Also, according to several studies, sleep deprivation and intense exercise cause an increase in lipid peroxidation (41, 42). MDA, the result of lipid peroxidation, is a reliable indicator of oxidative stress (15, 16). By comparing the sham and control groups, sleep deprivation increased the MDA and decreased the SOD levels significantly. All three doses of myrtle aqueous extract significantly decreased MDA levels compared to the control group and elevated SOD levels. This is in line with previous studies that demonstrated the effectiveness of medicinal plants in lowering MDA levels (16, 22, 35, 43). Consequently, the lower MDA and higher SOD activities in the treatment groups can reduce oxidative stress and fatigue caused by insufficient sleep.

Sleep deprivation increases the blood levels of inflammatory markers such as TNF-α and IL-1β (44, 45). Our control group exhibited increased TNF-α and IL-1β levels compared to the sham group, and the aqueous myrtle extract reversed these changes. TNF-α decreased in the 350, 700, and 1000 mg/kg treated groups at 35.6, 39.6, and 58.9 % of the control group. The highest decrease in IL-1β compared to the control group was seen in the 1000 mg/kg group, equal to 72.33% of the control group. Similar to our results, the cognitive abnormalities in mice with sleep deprivation (SD) were significantly improved by an aqueous extract of *Brassica rapa* L. Additionally, this extract significantly lowered serum levels of inflammatory factors and relieved SD-induced impairments in peripheral energy metabolism (46).

According to previous studies, central nervous system alterations induced by inflammatory cytokines (such as IL-1β and TNF-α) cause behavioral changes like fatigue and symptoms like depression (4, 47-49). Furthermore, negative emotions are another characteristic of central fatigue (39, 50). The open-field test is commonly regarded as a valid test for locomotor activity in rodents linked with negative emotions. In depressed, anxious, and exhausted rats, the parameters comprising total distance moved, duration in the center, and velocity decreased (39, 40). In line with previous studies, our findings indicate that sleep deprivation negatively affected the total distance moved, time in the center, and velocity in sleep-deprived groups. Myrtle extract at all three doses reversed these changes and improved the velocity and total distance, especially in the high-dose group. The time in the center was not changed significantly in the treatment groups compared to the control group.

Phytochemical studies of the myrtle fruit have confirmed the presence of several substances, such as phenolic compounds and flavonoids (29, 51). Considering the significance of oxidative stress and inflammation in fatigue, these substances can effectively reduce fatigue due to their antioxidant and anti-inflammatory effects (51). Also, the effectiveness of polyphenols in alleviating chronic fatigue has been proven (52). The flavonoids and polyphenols of *Abelmoschus esculentus* (L.) Moench (Okra) demonstrated anti-fatigue effects by reducing the level of MDA and increasing the level of SOD and glycogen reserves (53). Additionally, the rats' swimming time increased when exposed to the flavonoid quercetin-3-O-gentiobiose found in okra fruits which has antioxidant properties. This flavonoid increased liver glycogen stores, lowered TNF-α and IL-6 levels, and improved the antioxidant enzyme system by increasing glutathione peroxidase and SOD (35). The myrtle fruit also contains various compounds such as polysaccharides, tannins, anthocyanins, ascorbic acid, citric acid, and essential oil. The antioxidant and anti-inflammatory effects of anthocyanins and ascorbic acid have been proved by several studies which can be effective in reducing fatigue (54-56).

The results of previous studies showed the effectiveness of plant polysaccharides in reducing fatigue. Polysaccharides taken from plants such as *Polygnonatum odoratum, Changium smyrnioides, Ziziphus jujube*, and *Morinda emarginata* reduced fatigue in animal models by various mechanisms, including improving antioxidant system, reducing oxidative stress, increasing glucose levels, and changing inflammatory factors (16, 57-59). Since polysaccharides are a component of myrtle berries, they might contribute to the anti-fatigue effects observed in this study.

In conclusion, this study demonstrated that the aqueous extract of *M. communis* fruit reduced fatigue in two animal models, suggesting a therapeutic option for fatigued patients following adequate studies. The effectiveness of this plant in clinical trials should be investigated further.

## Data Availability

The corresponding author can provide the dataset upon request.
